# Donald Eccleston, FRCPsych, PhD, DSc

**DOI:** 10.1192/bjb.2018.60

**Published:** 2018-12

**Authors:** Ian McKeith

Formerly Professor of Psychiatry, Newcastle upon Tyne Medical School, UK


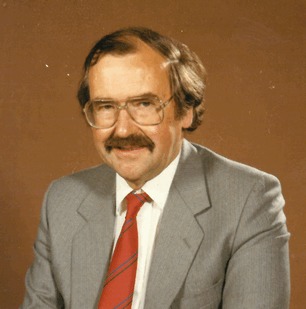


Donald (Don) Eccleston, who died recently at the age of 86 years, was one of the first to elaborate a hypothesis of depression which argued for the role of monoamines, particularly 5-hydroxytryptamine (5-HT), in the regulation of mood. He went on to translate his experimental observations into treatment for patients with refractory depressive disorders, devising the ‘Newcastle cocktail’ (phenelzine, L-tryptophan and lithium). This was a pharmacological strategy offering a window of opportunity through which cognitive–behavioural therapy (CBT) and intensive nursing care could be directed to reduce the secondary handicaps of chronic depression.

In 1962, he took up a post at the Medical Research Council (MRC) Brain Metabolism Unit at the University of Edinburgh, joining a highly innovative group of psycho-pharmacologists including George Ashcroft. Between them, over the next decade, they elaborated the role of amines, in particular 5-HT, in the regulation of mood. In the absence of today's sophisticated imaging and analytical techniques, these must have been difficult experiments to conduct; his first published paper describes measuring changes in 5-HT metabolites in volunteers who were being investigated for neurological disorders by air encephalography, a procedure in which most of the cerebrospinal fluid was drained from around the brain by means of a lumbar puncture and replaced with air.

In 1966, he spent an enjoyable year at the National Institutes of Health in Bethesda, Maryland, USA, where he worked with Julius Axelrod – one of the three winners who shared the 1970 Nobel Prize in Physiology or Medicine for their discovery of the actions of neurotransmitters in regulating the metabolism of the nervous system. He then returned to Edinburgh to be appointed Deputy Director of the MRC unit, where he continued to elaborate the monoamine hypothesis of depression by demonstrating that drugs which influence mood in humans may alter the turnover of 5-HT, and levels of 5-HT, in the brains of animals.

On appointment to the Chair of Psychiatry in Newcastle upon Tyne in 1977, he set up his own research unit at 1–4 Claremont Terrace and established the Regional Affective Disorders Unit, the longest-standing in-patient unit for the treatment of depression in the UK. The Newcastle Chronic Depression Study, published in 1987, described new therapeutic approaches to treatment-resistant chronic depression, including the use of CBT, which was just emerging from US research as having antidepressant potential. The Chronic Depression Study demonstrated a now well-accepted principle in the treatment of chronic depression, namely that intensive drug treatment may be a necessary preliminary that enables effective rehabilitation of the secondary handicaps. Donald promoted development of psychotherapeutic treatments, particularly CBT, and innovative nursing practices for his patients, and became increasingly interested in predictors of refractory depression and its prophylaxis.

While he was Head of the Academic Department of Psychiatry in Newcastle, lecturer and fellowship posts in his department were always in demand; it was a happy place to work, with many appointees going on to professorial appointments in psychiatry, psychology, nursing and neuroscience, both within and beyond Newcastle. He also trained a generation of research-supportive National Health Service (NHS) clinicians who, through him, had been exposed to evidence-based medicine just as the term began to be coined. Early on, he realised the limitations of an academic ivory tower. As the management of the NHS started to change, he recognised the mutual benefits of close partnerships between academia and care providers.

Born in Preston, Lancashire, Don was encouraged by his local general practitioner to consider a career in medicine, even if only to get enough money to support his preferred option of working on the land! He attended Preston Boys’ Grammar School. After leaving school, Don studied medicine in Aberdeen, balancing bookwork with what became lifelong interests: fishing, tennis and squash. In 1958, he married Maureen (Mo) Ellison and they settled in Aberdeen where he had commenced psychiatric training.

He was an early advocate of work–life balance, and this at a time when medical prowess was generally measured by how much time one spent at work. In addition to his family life, home and animals, Don made sure that he found plenty of time for his friends, his love of food and wine, fishing, squash, gardening and his art collection. His welcoming family home, West Luddick House, with its walled garden and greenhouses, was populated not only by Don and his family, but by generations of cats, dogs and hens. The monthly entry in his diary for ‘the Melrose Clinic’ was a euphemism for a day's fishing in the Scottish Borders and wasn't the most closely guarded secret from those around him.

After retirement in 1995, at the age of 64, Donald continued to see patients and remained involved in University life, but had more time to busy himself with his cottage deep in the Borders where he spent happy days foresting and looking after the land. He benefited from modern surgical advances, with a total of five joint replacements that kept him mobile. He died suddenly at home on 18 March 2018.

Don is survived by his wife Mo, three children and five grandchildren.

